# Brightness Perception in World-Centered Coordinates Assessed by Pupillometry

**DOI:** 10.3390/bs13010060

**Published:** 2023-01-09

**Authors:** Novera Istiqomah, Yuya Kinzuka, Tetsuto Minami, Shigeki Nakauchi

**Affiliations:** 1 Department of Computer Science and Engineering, Toyohashi University of Technology, Toyohashi 441-8580, Japan; 2 Department of Computer Engineering, Telkom University, Bandung 40257, Indonesia

**Keywords:** brightness perception, pupillometry, world-centered coordinates, cognition

## Abstract

Subjective brightness perception reportedly differs among the peripheral visual fields owing to lower- and higher-order cognition. However, there is still a lack of information associated with subjective brightness perception in the world-centered coordinates, not in the visual fields. In this study, we aimed to investigate the anisotropy of subjective brightness perception in the world-centered coordinates based on pupillary responses to the stimuli in five locations by manipulating the world-centered coordinates through *active* (requiring head movement) and *passive* scenes (without head movement) in a virtual reality environment. Specifically, this study aimed to elucidate if there is an ecological advantage in the five different locations in the world-centered coordinates. The pupillary responses to glare and halo stimuli indicated that the brightness perception differed among the five locations in the world-centered coordinates. Furthermore, we found that the pupillary response to stimuli at the top location might be influenced by ecological factors (such as from the bright sky and the sun’s existence). Thus, we have contributed to the understanding of the extraretinal information influence on subjective brightness perception in the world-centered coordinates, demonstrating that the pupillary response is independent of head movement.

## 1. Introduction

Different perceptions of an identical object located in the different eye visual fields (VFs) are known as VF anisotropy. VF anisotropy may be evoked by the opponent processes of many neural functions in the visual system. For example, the visual input signals projected onto the retina from the left VF are carried to the right primary visual cortex (visual area 1; V1) and vice versa. Furthermore, in human visual processing, the input signals from V1 are projected to the prestriate cortex (visual area 2; V2) via the ventral stream, representing visual input derived from the natural world.

In terms of a visual input representation, Andersen et al. (1993) proposed that the spatial information’s representation is configured by collecting visual stimuli information that is formed by various coordinate transformations during visual processing [1]. Furthermore, visual processing starts when the light rays hit the retina, and visual input signals are encoded in the retinal coordinates. Hereafter, the visual signals (retinal coordinates) are combined with the non-visual signals (extraretinal coordinates) in the brain to encode the visual stimuli. These extraretinal coordinates can be obtained from non-retinal coordinates. For example, first, head-centered coordinates refer to the head frame as the reference defined by integrating the retinal coordinates and position of the eye. Second, body-centered coordinates can be obtained by combining information regarding retinal, eye, and head positions. Third, world-centered coordinates are formed by collecting information of the head-centered coordinates and vestibular input (information source that senses the rotational movement for spatial updating).

In addition, in most recent studies focusing on perceptual differences among the VFs, the observers’ head was fixed, and the gaze was fixated on a reference object placed in the central VF. Many notable reports have been made on VF anisotropy (manipulating retinal coordinates) regarding many aspects of visual perception [2,3,4,5]. Specifically, the vertical hemifield has a dominant effect among the VFs compared with the horizontal hemifield [6]. Moreover, during psychophysical experiments that require attentional resources in response to a change in the light source, pupil sensitivity to light is higher in the upper visual field (UVF) than in the lower visual field (LVF) [7,8,9]. Additionally, objects located in the UVF are biased toward the extrapersonal region (for scene memory), whereas objects in the LVF are biased toward the peripersonal (PrP) region (for visual grasping) in 3D-spatial interactions. Other advantages of the LVF include better contrast sensitivity [10], visual accuracy [11], motion processing [12,13], and spatial resolution of attention and spatial frequency sensitivity [14]. The LVF bias in processing information about an object is caused by the substantially higher number (60% more) of ganglion cells in the superior hemiretina than in the inferior hemiretina [15], which results in an improved visual performance in the former.

VFs are also known to evoke different brightness perceptions. The perceptual brightness modulation is associated with cognitive factors such as memory and visual experience. This effect has been studied using pupillometry, with photographs and paintings of the sun as the stimuli. Binda et al. (2013) confirmed that sun photographs yielded a greater constriction of the pupils than did other stimuli despite physical equiluminant (i.e., squares with the same mean luminance as each sun photograph, phase-scrambled images of each sun photograph, and photographs of the moon) [16]. Subsequently, Castellotti et al. (2020) discovered that paintings including a depiction of the sun produce greater pupil constriction than paintings that include a depiction of the moon or no depiction of a light source, despite having the same overall mean luminance [17]. Recently, Istiqomah et al. (2022) reported that pupillary response to the image stimuli perceived as the sun yielded larger constricted pupils than those perceived as the moon under average luminance-controlled conditions [18]. Their results indicated that perception has a dominant role rather than a mere physical luminance of the image stimuli due to the influence of ecological factors such as the existence of the sun. All of these studies demonstrate that pupillometry reflects not just the physical luminance (low-order cognition) but also the subjective brightness perception (higher-order cognition) in response to the stimuli. In addition, the previous study by Tortelli et al. (2022) confirmed that pupillary response was influenced by contextual information (such as from the sun’s images) considering the differences of inter-individual differences in the observer’s perception [19].

Pupillometry is a metric used to measure pupil size in response to stimuli and may reflect various cognitive states. The initial change in pupil diameter is caused by the pupillary light reflex (PLR). However, the degree of change in pupil diameter is influenced by visual attention, visual processing, and the subjective interpretation of brightness. For example, Laeng and Endestad (2012) reported that a glare illusion conveyed brighter than its physical luminance induced greater constricted pupils [20]. This glare illusion has a luminance gradient converged toward the pattern’s center that enhances the brightness intensely [21,22]. Furthermore, Laeng and Sulutvedt (2014) revealed that, owing to the response of the eyes to hazardous light (such as sunshine), the pupils considerably constricted when the participant imagined a sunny sky or the face of their mother under the sunlight [23]. Other previous study by Mathôt et al. (2017) revealed that words conveying a sense of brightness yielded a greater constriction of pupils than those conveying a sense of darkness [24]. These differences indicated the pupils’ response to a source that may damage the eyes despite only occurring in the observer’s imagination. In addition, Suzuki et al. (2019) revealed that the pupillary response to the blue glare illusion generated the largest pupil constrictions, reporting that blue is a dominant color in the human visual system in natural scenes (e.g., the blue sky) and indicating that, despite the average physical luminance of glare and control stimuli being identical, pupillary responses to the glare illusion reflect the subjective brightness perception [22].

Recently, we demonstrated that the pupillary response to glare and halo stimuli differed depending on whether the stimuli were presented in the upper, lower, left, or right VFs by manipulating the retinal coordinates [25]. We found that pupillary responses to the stimuli (glare and halo) in the UVF resulted in the largest pupil dilation and significantly reduced pupil dilation, specifically in response to the glare illusion due to higher-order cognition. The previous results reflect that the glare illusion was a dazzling light source (the sun) influencing the pupillary responses. However, our previous study and other studies regarding the subjective brightness perception analysis in the VFs (also mentioned in paragraph 3) raise the possibility that the differences in retinal coordinates and many opponent processes in the human visual system will affect the subjective brightness perception in the VFs. Therefore, clarifying whether there is anisotropy of subjective brightness perception by maintaining identical retinal coordinates and manipulating the world-centered coordinates could provide valuable insights into the anisotropy of subjective brightness perception in the world-centered coordinates based on pupillary responses to the glare illusion and halo stimuli. Particularly, this study aimed to elucidate whether there is an ecological advantage in five different locations in the world-centered coordinates based on pupillary responses to the glare illusion overtly that conveys a dazzling effect.

The difference between our previous and present studies is the visual input, which used the retinal coordinates manipulation in our previous study, and world-centered coordinates (formed by collecting information of the head-centered coordinates and vestibular input) manipulation in this work. To investigate the anisotropy of subjective brightness perception in the world-centered coordinates, we presented the glare and halo as stimuli in five different locations (top, bottom, left, right, and center) in the world-centered coordinates based on the pupillary responses to the stimuli (glare and halo) while the observers fixated on a fixation cross located in the middle of the stimulus. We used a virtual environment to easily control the physical luminance of the stimuli and the designated environment. In addition, the contextual cues of the 3D virtual environment provide more cues of features associated with the given tasks and advantages in decreasing the visual perception area; thus, the observers would perceive the stimuli easily [26]. Furthermore, to form the world-centered coordinates, adding vestibular input to be combined with head-centered coordinates (retinal coordinates and eye position integration) is required. Therefore, we adopted an *active* scene that instructed the observers to move their heads in accordance with the stimulus’ location in the world-centered coordinate as the vestibular input. To ensure that the present study’s results are not merely pupil size artifacts induced by the head movement during the *active* scene, we manipulated the scene by automatically moving the virtual environment as the substance of the head movement in the *active* scene, called the *passive* scene, which did not allow the head movement during the stimulus presentation. In addition, we also applied glare as the stimuli and halo manipulation as the stimuli to find out whether there is any distinction between pupillary responses to the glare and halo stimuli, particularly, associated with ecological factors, as the representation of the sun [22,25], in five locations in the world-centered coordinates. In the present study, through an *active* and *passive* scene, we hypothesized that there is anisotropy in the pupillary responses in the world-centered coordinates; particularly, the results would generate the highest difference between pupillary responses to the glare (more constrict than halo) and halo stimuli at the top, and pupillary responses to the stimuli at the top would yield the highest degree of pupillary constriction as a consequence of ecological factors such as avoiding the dazzling effect of sunshine entering the retina.

## 2. Materials and Methods

### 2.1. Participants

A total of 20 participants (15 men and 5 women, aged between 23 and 35 years; mean age = 27.1 and SD = 4.04 years) participated in this study. Two observers’ data regarding the change in pupil size were excluded from the analyses as the trial rejection ratio did not exceed 30% after interpolation and filtering in the pre-processing stage. All participants had a normal or corrected-to-normal vision. All experimental procedures were conducted according to the ethical principles outlined in the Declaration of Helsinki and approved by the Committee for Human Research at our university. The experiment was conducted with complete adherence to the approved guidelines of the committee. Written informed consent was obtained from the participants after procedural details had been explained to them.

### 2.2. Stimuli and Apparatus

We conducted two experiments on each observer, i.e., the *active* and *passive* scenes in the VR environment. We used Tobii Pro VR Integration, which has an eye-tracker installed in HTC Vive HMD, to present the stimuli. We measured pupil diameter and eye gaze movements using an infrared camera at a sampling rate of 90 Hz. As the output, the device produced pupil diameter in meter. We developed the VR environment by using the Unity version 2018.4.8f1 game engine. The HTC Vive HMD has a total resolution of 2160 × 1200 pixels on two active-matrix organic light-emitting diode screens and a 110° field of view.

The pupil size data measured by the Tobii Pro were transferred to Unity to be saved and processed with the stimulus presentation data. The observer’s location in the VR environment was in the center of the gray-grid-sphered background developed in Blender 2.82 software (open-source software for 3D computer graphics). The gray-grid-sphered background was used to provide a sign that the VR environment moved when the observer moved their head.

Moreover, we conducted two experiments through the *active* scene, in which the observer needed to move their head according to the location of the stimulus in the VR environment, and the *passive* scene, in which the observer needed to keep their head stable during the experiment. For the *passive* scene, we recorded the head movement coordinates of four people in a preliminary study using the HTC Vive Pro Eye HMD with an identical VR environment and refresh rate of 90 Hz. Each recording was played to the participants as a replacement for their head movements. By reproducing the head movement coordinates, the VR environment moved automatically according to the location of the stimuli during the experiment. Detailed information on the flow of the experiments is presented in the Procedure subsection.

**Figure 1:**
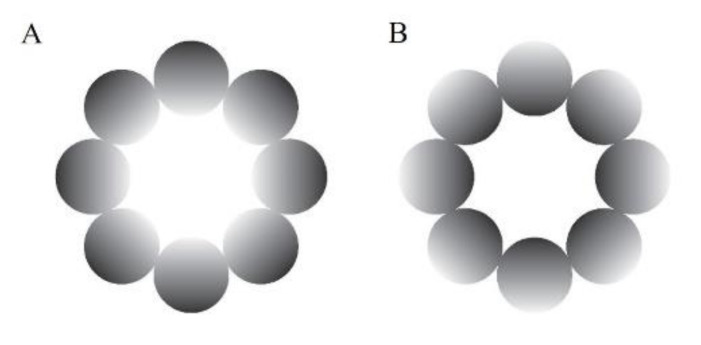
Experimental stimuli. (**A**) The glare illusion, with an increasing luminance gradation from the periphery to the central white region; (**B**) the halo stimulus, with a decreasing luminance gradation from the periphery to the center.

An achromatic glare illusion (Figure 1A), in which the luminance gradation increases from the periphery to the central white region, and a halo stimulus (Figure 1B), in which the luminance gradation diverges from the periphery to the center, were presented as the stimuli in this study. We used these types of illusion because they have many advantages over the Asahi and ring-shaped glare illusions (Istiqomah, et al., 2022), such as easily distributing the stimulus’ physical luminance evenly in the retina compared with the Asahi and ring-shaped glare illusions, creating its inverse form, and ensuring that the average physical luminance between the glare and its inverse form (halo) was the same. In the gray-grid-sphered background, we used the RGB colors [130, 130, 130] and [100, 100, 100] for the gray circle and fixation cross, respectively. Furthermore, for the unit of detailed stimuli and VR environment, we used the Unity unit (one Unity unit identical to one meter). The distance between the participant and the stimulus in the VR environment was 100 m. The stimuli comprised eight luminance gradation circles, each positioned with its center 14.41 m from the center of the stimulus (approximate visual angle of 8.24°), and each gradation circle’s diameter was 11.19 m (approximately 6.40°). The central white area of the stimulus was 17.62 m in diameter (approximately 10.07°). Therefore, the overall stimulus diameter was 40 m (approximately 22.62°). The fixation cross was 2.93 m in diameter (approximately 1.68°). The stimuli presented at the VR environment’s top, bottom, left, and right were tilted 76.64 m from the central position (approximately 65°). In addition, we analyzed the pupillary size data using MATLAB R2021a.

### 2.3. Procedure

**Figure 2:**
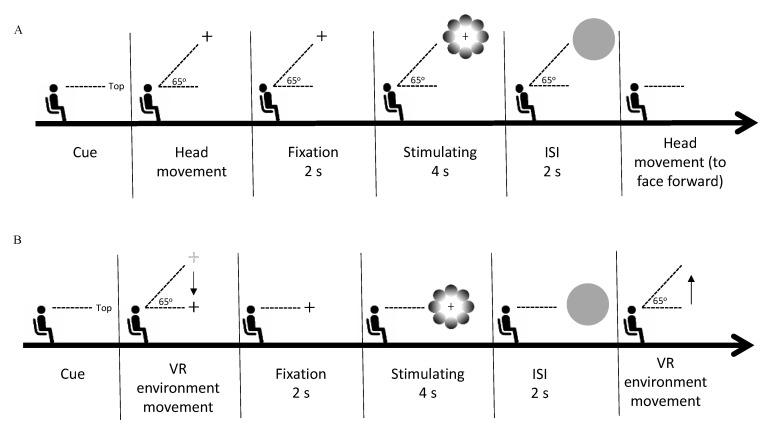
Experimental procedure. (**A**) The phase sequence of one trial in the *active* scene. Each trial started with the presentation of a textual cue for the direction of the stimulus, following which the observer moved their head in the indicated direction. Next, the observer fixated on the fixation cross for 2 s (fixation phase). Thereafter, a random stimulus (glare/halo) was presented for 4 s (stimulus presentation phase), during which the observer had to keep fixating on the fixation cross without blinking their eyes. Subsequently, the observer was requested to keep their head stable during the 2-s presentation of a gray circle (interval phase). In the last phase, the observer moved their head back to the original position to face forward. (**B**) In the *passive* scene, the procedure of one trial was the same as in the *active* scene, except that the observer was not allowed to move their head. After the directional cue was presented, the VR environment automatically moved in the indicated direction. In the last phase, the VR environment moved back to its original location to substitute the head movement to face forward. The automated VR environment movement occurred by playing the coordinates of prerecorded head movements.

We were able to produce the same retinal coordinates through the *active* and *passive* scenes by placing the stimulus in the five locations of the VR environment and instructing the observer to fixate their gaze on the fixation cross located in the stimulus center, corresponding to world-centered coordinates. In the *active* scene, participants were required to move their heads, whereas, in the *passive* scene, the recording of head movement coordinates displaced the head movement toward the stimulus location. We measured the pupil diameter in response to the stimuli in accordance with the stimulus’ location during the stimuli presentation. Both experiments (*active* and *passive*) were conducted with the observer in the sitting position and facing forward. The experiments were conducted on different days randomly to prevent eye fatigue caused by the first experiment from influencing the pupillary response in the second experiment. We calibrated the integrated eye tracker on the HMD by performing a standard, five-point calibration before the beginning of each session. In the *active* scene, each trial started with a direction text presentation of the stimulus locations, appearing in the center of the observer’s VF. The observer was instructed to move their head in the direction indicated by the text prompt (top, bottom, left, right, or center, in random order), where they would find the fixation cross. After fixating on the fixation cross for two seconds, the observer was presented with a random stimulus (glare or halo), and the fixation cross remained in the center of the stimulus for four seconds. In the next stage, a gray circle appeared for two seconds to neutralize the observer’s pupil size. The observer had to keep their head stable until the gray circle disappeared. Thereafter, the observer reoriented their head to face forward. The procedure for the *passive* scene was the same as that for the *active* scene, except that the observer was instructed not to move their head, as the VR environment would automatically move in the direction indicated by the text prompt by playing the recording of the preliminary study (see the Stimuli and apparatus subsection). Details of the procedures in the present study are provided in Figure 2. In each experiment, each stimulus (glare and halo) was presented 15 times per location (top, bottom, left, right, and center). Thus, each experiment consisted of 150 trials (5 locations × 2 gradient patterns × 15 trials), including two breaks of approximately 15 min each, and the session after the break started with the eye-tracker calibration.

### 2.4. Pupil and Eye Gaze Analyses

**Figure 3:**
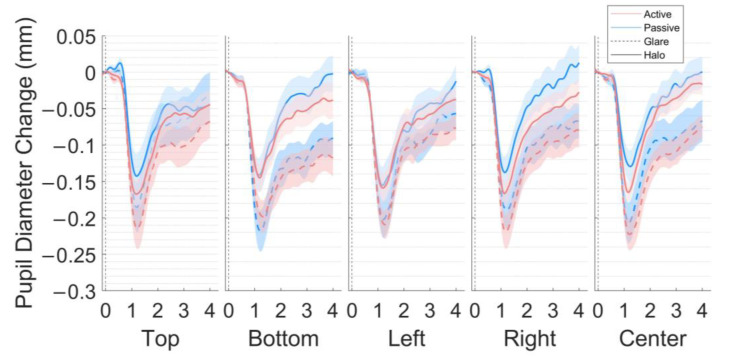
Pupil size changes in the five locations (top, bottom, left, right, and center) in the world-centered coordinates. The grand average of pupillary response to the glare illusion and halo stimulus (millimeters) during the 4-s stimulus presentation for 18 participants after subtractive baseline correction in the *active* and *passive* scenes. The dotted line represents the baseline period (−0.2 s).

We used cubic Hermite interpolation for the pupil, and eye gaze data during eye blinks displayed as “NaN” values for the pupil data and zero values for the gaze data. Thereafter, we applied the subtractive baseline correction by calculating the mean of 0.2 s pupillary responses before the stimulus onset to define the baseline and subtracting the pupil size from the baseline in each trial (the dotted line in Figure 3 represents the baseline period). Furthermore, a low-pass filter for data smoothing with a 4-Hz cut-off frequency was implemented, as in a previous study [27]. The analysis excluded data from trials with additional artifacts, calculated by thresholding the peak changes on the velocity of change in pupil size (more than 0.001 mm/ms). In addition, the trials were rejected with a Euclidian distance (calculated using the first and second principal components) exceeding 3 σ of all trials. After that, we also rejected the trials if the average of eye gaze fixation during the stimuli presentation exceeded the radius of 5.035° (i.e., the central white area of the stimulus). In the last stage of preprocessing data, we rejected two participants due to the rejected trials ratio exceeding 30%. The average rejection ratios were 14.20% and 1.7% of all trials per observer in the *active* and *passive* scenes, respectively. We applied this preprocessing procedure to pupil and eye gaze data.

In addition, for pupil diameter data, we separated the data into two approaches, early and late components [25,28,29].

(1) The early component reflected pupillary responses modulated by the physical luminosity of the stimuli via low-order cognition. First, we calculated the pupil slope using second-order accurate central differences to attain the maximum pupil constriction latency (MPCL) of the series data from the beginning of the stimulus presentation until 1 s, which accommodated the large pupil diameter change triggered by the PLR, in each trial and participant (the exact procedure with our previous work to obtain MPCL values, [25]). Thereafter, we grand averaged the pupil data using the following function: $\bar{X_{MPCL\pm 0.1}}$, where x shows the pupil size at approximately 0.1 s before and after the MPCL as the early component (in millimeters, mm).

(2) The late component (using area under curve, AUC) was significantly influenced by emotional arousal as well as subjective brightness perception via higher-order cognition [25,28,29]. Furthermore, the late component represented the pupil diameter in more time to come back to its initial state, which was calculated as follows:

(1) $$AUC=\overset{4}{\underset{i=\mathrm{MPCL}}{\sum }}x_{i}-x_{\mathrm{MPCL}}$$

where x represents the pupil diameter at i seconds when the MPCL occurred until stimulus offset at 4 s. We applied this function to all series data of pupil size in each trial and observer. In the last step, we grand-averaged the size data across the trials and observers for each stimulus pattern and location (in the unit of mm).

### 2.5. Statistical Analyses

We used three-way repeated-measures (rm) analysis of variance (ANOVA) to compare the pupillary responses and *y*-axes of eye gaze data between the *active* and *passive* scenes. The rmANOVA conditions were as follows: two scenes (*active* and *passive*), five stimulus locations (top, bottom, left, right, and center), and two stimulus patterns (glare illusion and halo stimulus). We used Greenhouse–Geisser correction when Mauchly’s sphericity test revealed significant differences between the variances of the differences. For the main effect and post-hoc pairwise comparisons, *p*-values were corrected with the Holm–Bonferroni method, and the resultant significance level (α) was set at <0.05 for all analyses. Cohen’s d and the partial $\eta^{2}$ ($\eta_{p}^{2}$) were used to represent effect sizes [30]. All statistical analyses were performed using JASP version 0.16.4.0 software [31]. Additionally, we also performed a Bayesian rmANOVA analysis using JASP with default priors, and the BF_M_ and BF_10_ represent the effect in the model comparison and post hoc comparison by only considering ‘matched models’ due to a more conservative assessment than ‘across all models’, and ‘compared to best model’ as the ‘Order’ [32]. We used the recommendation of Jeffreys (1961) as the guidelines for Bayes factor interpretation [32].

## 3. Results

**Figure 4:**
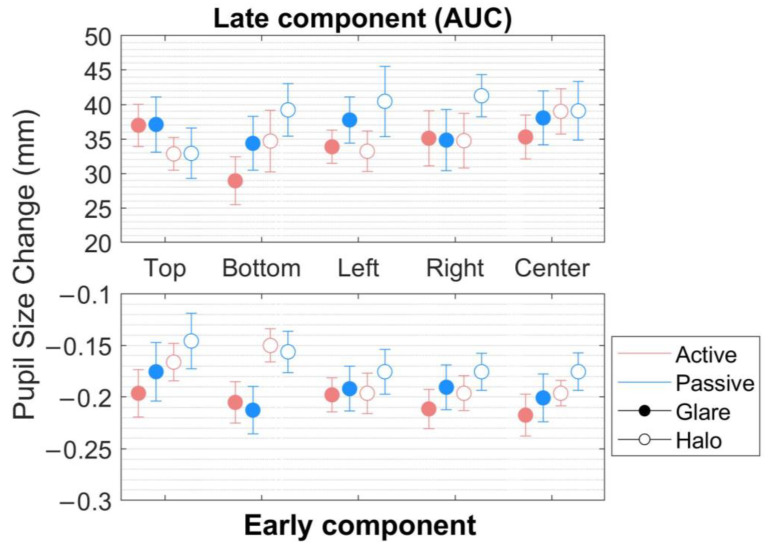
Pupillary response to the glare and halo stimuli in the early component. Pupil diameter changes (mm) in five locations (top, bottom, left, right, and center) in the world-centered coordinates for 20 participants in the *active* and the *passive* scenes. Error bars indicate standard errors of the mean.

The main results of the present study are presented as the pupil size and *y*-axis of eye gaze in response to the glare and halo stimuli for four seconds across the five locations in each scene. The time courses of the pupillary responses to each stimulus pattern (glare and halo), stimulus location (top, bottom, left, right, and center), and scene (*active* and *passive*) are illustrated in Figure 3 (4-s exposure). We separated the pupil size data, based on the MPCL value), i.e., early and late components (Figure 4).

**Figure 5:**
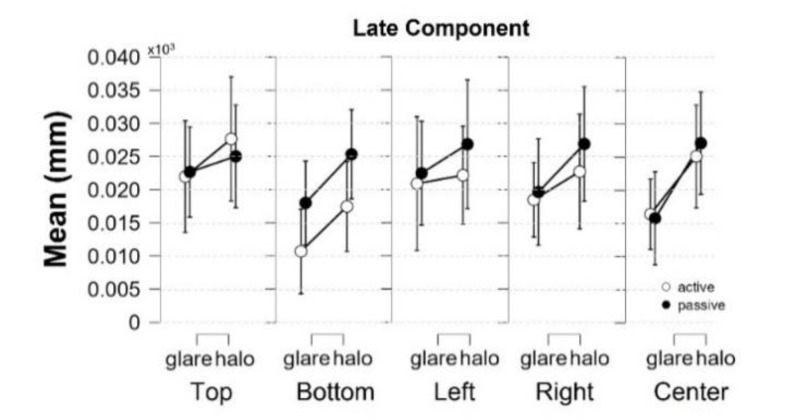
Descriptive plots of Bayesian rmANOVA in the late component. The mean of pupil diameter change (mm) in response to the stimuli at the bottom was the smallest value compared with the other locations in the world-centered coordinates. Error bars indicate lower and upper values with a 95% credible interval.

(1) In the early component (Figure 4, bottom), within the range of around 0.1 s before and after MPCL value, an rmANOVA of the pupillary response to the stimuli revealed very strong evidence for the presence of stimulus pattern (*F*[1,17] = 58.899, *p* < 0.001, $\eta_{p}^{2}$ = 0.776, BF_M_ = 90.205) but not of the scene, location, and no interaction effect between the parameters (scene, stimulus pattern, and location) (Table 1 and Table 2). 

(2) In the late component (the area under the curve [AUC]) (Figure 4, top), defined as integral values of pupillary responses from MPCL value to the end of the stimulus presentation, three-way rmANOVA revealed strong evidence for the presence of a stimulus pattern (*F*[1,17] = 12.437, *p* = 0.003, $\eta_{p}^{2}$ = 0.423, BF_M_ = 26.005), and a significant main effect on location (*F*[2.944,50.044] = 3.469, *p* = 0.023, $\eta_{p}^{2}$ = 0.169, BF_M_ = 0.019) (Table 3 and Table 4). Nevertheless, the post hoc comparisons on location (from the classical frequentist), the Bayesian rmANOVA on location, and other conditions neither show a significant effect. Moreover, further investigation on the post hoc comparison of location from Bayesian analysis obtained moderate evidence only in pairs of top-bottom (*t*[18] = 2.586, *p* = 0.192, Cohen’s *d* = 0.312, BF_10,U_ = 6.660) and bottom-left (*t*[18] = −2.927, *p* = 0.094, Cohen’s *d* = −0.251, BF_10,U_ = 3.469). Additionally, we plotted the descriptive information of Bayesian rmANOVA (Figure 5), and the results indicated that the pupillary response to the stimuli at the bottom location has the smallest mean of pupil size change in AUC compared with other conditions. 

Finally, we conducted a three-way rmANOVA (5 locations × 2 stimulus patterns × 2 scenes) on the *y*-axis of the eye gaze data to verify that the retinal coordinates were identical across the stimulus locations and patterns between the scenes. We found moderate evidence in favor of the stimulus patterns (*F*[1,17] = 4.195, *p* = 0.056, $\eta_{p}^{2}$ = 0.198, BF_M_ = 6.845) (Table 5 and Table 6). However, there was neither evidence in the post hoc comparison of stimulus patterns in the Bayesian rmANOVA.

## 4. Discussion

Our previous study reported that the peripheral VFs (upper, lower, left, and right) in which the glare and halo stimuli were located influenced the subjective brightness perception of participants, as represented by the pupillary response to those stimuli [25]. The UVF generated a greater pupil dilation in response to either stimulus than did the other VFs, and reduced pupil dilation in response to the glare illusion than that in response to the halo stimulus. The results were attributed to higher-order cognitive bias formed by statistical regularity in the processing of natural scenes. However, in our previous study’s results, it is possible that the differences in retinal coordinates would affect pupil size. The pupillary responses to the stimuli were influenced by pupil sensitivity, spatial resolution, and brightness perception (lower-order cognition) [7,14,33]. Therefore, to further investigate subjective brightness perception, not only in the peripheral VFs (our previous study’s results), we conducted experiments through *active* and *passive* scenes by maintaining identical retinal coordinates and manipulating the world-centered coordinates, that is, by presenting the glare and halo as the stimuli in five different locations (top, bottom, left, right, and center) in the VR environment to investigate the anisotropy of subjective brightness perception in the world-centered coordinates. By manipulating the world-centered coordinates, we confirmed that the pupillary responses in each location differed despite the retinal coordinates being identical.

Furthermore, we divided the pupil size data into two components based on the MPCL values, that is, the early component, to evaluate the pupillary responses induced by the PLR around the area of 0.1 s before to after MPCL value, and the late component (the AUC), to access higher-order cognition (e.g., emotional arousal and subjective brightness perception) using Function 1 [25,28,29]. 

(1) The early component. Our data provide very strong evidence for the presence of stimulus patterns (*F*[1,17] = 58.899, *p* < 0.001, $\eta_{p}^{2}$ = 0.776, BF_M_ = 90.205). The significantly constricted pupil in response to the glare compared to halo stimuli reflects the enhancement of perceived brightness [20]. In previous studies, the pupillary responses, especially during the PLR period, revealed the alteration of physical light intensity by means of lower-level visual processing [21,34]. The PLR is elicited by visual attention, visual processing and interpretation of the visual input [34] and, possibly, higher-order cognitive involvement [35]. Hence, the low-order cognition (enhancement of brightness perception) may affect the pupillary response in the early component, as evoked by the enhancement in brightness perception. However, the early component analysis in the present study was insufficient. It had not yet fulfilled the present work’s aim to elucidate whether there is an ecological advantage in the five different locations in the world-centered coordinates, which belong to high-level visual processing.

Therefore, we further investigated the pupillary response in the late component. 

(2) Late component (AUC). The presence of stimulus pattern generated strong evidence (*F*[1,17] = 12.437, *p* = 0.003, $\eta_{p}^{2}$ = 0.423, BF_M_ = 26.005) in the effect of stimuli’s physical light intensity entered the retina (low-order cognition) after the minimum peak of pupil response (MPCL). This evidence might be neither merely induced by the physical luminance of glare and halo stimuli, yet also indicated the complex visual processing.

Furthermore, our data show a significant main effect in location (*F*[2.944,50.044] = 3.469, *p* = 0.023, $\eta_{p}^{2}$ = 0.169, BF_M_ = 0.019). We were further investigating the post hoc comparison of location from the classical frequentist rmANOVA, and there were no significant effects in any pairs of locations. In line with the previous study by Keysers et al. (2020), we used the Bayesian factor hypothesis to overcome the absence of evidence in the post hoc comparison of location from the classical frequentist rmANOVA [36]. Considering the Bayesian factor hypothesis, the post hoc comparisons on location generated moderate evidence in the pairs of top-bottom (*t*[18] = 2.586, *p* = 0.192, Cohen’s *d* = 0.312, BF_10,U_ = 6.660) and bottom-left(*t*[18] = −2.927, *p* = 0.094, Cohen’s *d* = −0.251, BF_10,U_ = 3.469). Moreover, descriptive plots generated by JASP (Figure 5) exhibit the smallest mean of pupil size change in response to the stimuli at the bottom. Contrary to our hypothesis that the pupil would be most constricted in response to the stimuli at the top, we demonstrated that the response to the stimuli at the bottom obtained a higher degree of pupil constriction than the stimuli at the top location.

The highest degree of pupil constriction produced by the pupillary response to the stimuli at the bottom was linked to one of four areas in the 3D-spatial interactions model theory proposed by Previc (1998) [37]. One of those areas is the region in which a person can easily grasp items (such as edible objects for consumption), known as the PrP region. The PrP region has a lower field bias within a 2-m radius from the observer. Objects that have already been observed are processed in the PrP region. Furthermore, the PrP region in the virtual environment, especially as the first person (FP) without an extended part of the FP (as we did in the present work), is defined by the peripheral space of the FP. It will have a large field of visual perception compared to the extended PrP region and no visual obstacle [38]. Therefore, visual processing (recognition and memorization) of objects in the PrP region requires minimal effort (an easier task for an observer’s eyes). The low demand for responses to stimuli presented at the bottom in world-centered coordinates resulted in a higher degree of pupil constriction than that in response to stimuli presented at the top. In addition, statistical analysis of pupil data in the present study revealed no significant main effect of the scene in either the early or late component. This result confirmed that the head movement did not affect the pupillary response during the stimulus onset.

Considered together, the complex visual processing induced by the glare and halo stimuli and the moderate evidence from the Bayesian factor, particularly in the pair of top-bottom locations, in the late component implies that the subjective brightness perception represented by the pupillary responses to the stimuli at the top in the world-centered coordinates might be influenced by the ecological factors. For instance, first, the ecological factor evoked by the glare and halo stimuli due to the glare illusion in the present study represents the sun [22,25]. Second, the stimuli at the top were perceived as darker than those at the bottom due to the cognitive bias related to the natural scenery where the bright blue sky is present [22]. All the evidence in our study demonstrates anisotropy of subjective brightness perception among the five locations in the world-centered coordinates. These differences in subjective brightness perception occurred even though we applied the same stimulus luminance and the same retinal coordinates across the five locations due to extraretinal information tied to the ecological factors. Moreover, the *y*-axis gaze angle did not seem to affect the pupil diameter, indicating identical retinal coordinates. For future studies, presenting different stimuli (e.g., the ambiguous sun and moon images) and asking the observer’s perception whether the stimuli perceived as the sun or moon should be conducted to fully segregate the low-order cognition involvement on pupillary response to the stimuli.

We have two limitations in the present study. First, the eye rotation during the experiment (foreshortening with gaze angle) may have influenced the pupil size measurements in this study owing to the HMD being integrated with cameras that are used to record eye movements. We attempted to minimize this limitation during the experiment by instructing the participants to fixate on the fixation cross. Furthermore, we rejected trials based on the fixation of the eye gaze. Second, we considered only the vertical field of world centered-coordinates due to the fact that we would elucidate whether the ecological factors (such as from the sun’s existence) affect the subjective brightness perception in the world-centered coordinates. Thus, we believe that the present study offers valuable insights into the anisotropy of subjective brightness perception among the five locations (top, bottom, left, right, and central) in the world-centered coordinates, especially to understand the extraretinal information influence on subjective brightness perception in the world-centered coordinates, as revealed by using the glare illusion, manipulating the world-centered coordinates in a VR environment, and performing pupillometry. In addition, the present study provides valuable insight into the ophthalmology field that the pupillary response is not affected by head movement.

## 5. Conclusions

In the present study, we conducted the experiment by presenting the stimuli and manipulating the world-centered coordinates (top, bottom, left, right, and center) in a VR environment through *active* and *passive* scenes based on pupillary response to the glare and halo. We found anisotropy of subjective brightness perception among the five locations in the world-centered coordinates due to extraretinal information triggered by the ecological factors. In addition, we confirmed the independence of head movement in pupil diameter. In future studies, showing different stimuli (e.g., the ambiguous sun and moon images) and asking the observer’s perception whether the stimuli perceived as the sun or moon should be conducted to fully segregate the low-order cognition in our results on pupillary response to the stimuli should be conducted.

## Figures and Tables

**Table 1:** The main effect of three-way rmANOVA in the early component.

Effects	df	*F*	*p*	*η^2^p*
scene	1.000	0.034	0.855	0.002
pattern	1.000	58.899	<0.001	0.776
location	2.430	2.103	0.126	0.110
scene × pattern	1.000	1.368	0.258	0.074
scene × location	2.931	2.003	0.127	0.105
pattern × location	2.677	0.810	0.483	0.045
scene × pattern × location	3.064	0.483	0.700	0.028

**Table 2:** Model comparison using Bayesian rmANOVA in the early component.

Models	P(M)	P(M|Data)	BF_M_	BF_10_	Error %
pattern	0.053	0.834	90.205	1.000	
Scene + pattern	0.053	0.121	2.483	0.145	2.028
Scene + pattern + Scene × pattern	0.053	0.027	0.493	0.032	2.595
pattern + location	0.053	0.015	0.279	0.018	1.753
Scene + pattern + location	0.053	0.002	0.041	0.003	1.875
Scene + pattern + location + Scene × pattern	0.053	4.728 × 10^−4^	0.009	5.672 × 10^−4^	2.286
pattern + location + pattern × location	0.053	3.294 × 10^−4^	0.006	3.951 × 10^−4^	2.715
Scene + pattern + location + Scene × location	0.053	7.812 × 10^−5^	0.001	9.371 × 10^−5^	2.651
Scene + pattern + location + pattern × location	0.053	4.981 × 10^−5^	8.966 × 10^−4^	5.975 × 10^−5^	4.499
Scene + pattern + location + Scene × pattern + Scene × location	0.053	1.853 × 10^−5^	3.335 × 10^−4^	2.223 × 10^−5^	9.561
Scene + pattern + location + Scene × pattern + pattern × location	0.053	1.152 × 10^−5^	2.074 × 10^−4^	1.382 × 10^−5^	8.862
Scene + pattern + location + Scene × location + pattern × location	0.053	1.628 × 10^−6^	2.930 × 10^−5^	1.953 × 10^−6^	2.833
Scene + pattern + location + Scene × pattern + Scene × location + pattern × location	0.053	3.526 × 10^−7^	6.346 × 10^−6^	4.229 × 10^−7^	3.111
Scene + pattern + location + Scene × pattern + Scene × location + pattern × location + Scene × pattern × location	0.053	1.671 × 10^−8^	3.008 × 10^−7^	2.004 × 10^−8^	3.190
Null model (incl. subject)	0.053	1.534 × 10^−8^	2.761 × 10^−7^	1.840 × 10^−8^	1.172
Scene	0.053	2.205 × 10^−9^	3.969 × 10^−8^	2.645 × 10^−9^	2.138
location	0.053	2.526 × 10^−10^	4.547 × 10^−9^	3.030 × 10^−10^	1.397
Scene + location	0.053	3.690 × 10^−11^	6.643 × 10^−10^	4.427 × 10^−11^	1.888
Scene + location + Scene × location	0.053	1.153 × 10^−12^	2.076 × 10^−11^	1.384 × 10^−12^	2.079

**Table 3:** The main effect of a three-way rmANOVA in the late component.

	df	*F*	*p*	*η^2^_p_*
scene	1.000	0.268	0.612	0.016
pattern	1.000	12.437	0.003	0.423
location	2.944	3.469	0.023	0.169
scene × pattern	1.000	0.194	0.665	0.011
scene × location	2.509	1.183	0.323	0.065
pattern × location	2.370	1.551	0.222	0.084
scene × pattern × location	3,476	0.381	0.795	0.022

**Table 4:** Model comparison using Bayesian rmANOVA in the late component.

Models	P(M)	P(M|Data)	BF_M_	BF_10_	Error %
pattern	0.053	0.591	26.005	1.000	
Scene + pattern	0.053	0.243	5.767	0.411	3.596
pattern + location	0.053	0.072	1.390	0.121	2.012
Scene + pattern + Scene × pattern	0.053	0.039	0.729	0.066	2.610
Scene + pattern + location	0.053	0.029	0.534	0.049	2.257
Null model (incl. subject)	0.053	0.010	0.174	0.016	1.546
Scene + pattern + location + Scene × pattern	0.053	0.005	0.093	0.009	3.534
Scene	0.053	0.004	0.065	0.006	1.794
pattern + location + pattern × location	0.053	0.003	0.053	0.005	2.218
Scene + pattern + location + Scene × location	0.053	0.002	0.043	0.004	30.011
Scene + pattern + location + pattern × location	0.053	0.001	0.022	0.002	2.722
location	0.053	0.001	0.019	0.002	1.607
Scene + location	0.053	4.200 × 10^−4^	0.008	7.107 × 10^−4^	2.787
Scene + pattern + location + Scene × pattern + Scene × location	0.053	3.108 × 10^−4^	0.006	5.260 × 10^−4^	6.270
Scene + pattern + location + Scene × pattern + pattern × location	0.053	2.234 × 10^−4^	0.004	3.781 × 10^−4^	6.914
Scene + pattern + location + Scene × location + pattern × location	0.053	6.962 × 10^−5^	0.001	1.178 × 10^−4^	3.157
Scene + location + Scene × location	0.053	2.189 × 10^−5^	3.940 × 10^−4^	3.704 × 10^−5^	1.888
Scene + pattern + location + Scene × pattern + Scene × location + pattern × location	0.053	1.308 × 10^−5^	2.354 × 10^−4^	2.213 × 10^−5^	9.265
Scene + pattern + location + Scene × pattern + Scene × location + pattern × location + Scene × pattern × location	0.053	6.316 × 10^−7^	1.137 × 10^−5^	1.069 × 10^−6^	3.348

**Table 5:** The main effect of three-way repeated measures ANOVA in *y*-axis gaze data.

	df	*F*	*p*	*η^2^_p_*
scene	1.000	0.157	0.697	0.009
pattern	1.000	0.480	0.498	0.027
location	2.705	2.749	0.059	0.139
scene × pattern	1.000	0.406	0.533	0.023
scene × location	2.854	2.088	0.117	0.109
pattern × location	3.087	0.842	0.480	0.047
scene × pattern × location	3.106	0.369	0.783	0.021

**Table 6:** Model comparison using Bayesian rmANOVA in *y*-axis gaze data.

Model Comparison					
Models	P(M)	P(M|Data)	BF_M_	BF_10_	Error %
Null model (incl. subject and random slopes)	0.053	0.566	23.507	1.000	
pattern	0.053	0.276	6.845	0.486	8.861
location	0.053	0.075	1.467	0.133	3.666
pattern + location	0.053	0.032	0.593	0.056	2.686
Scene	0.053	0.026	0.489	0.047	98.912
Scene + location + Scene × location	0.053	0.016	0.293	0.028	93.708
Scene + pattern + location + Scene × location	0.053	0.003	0.056	0.005	68.789
pattern + location + pattern × location	0.053	0.002	0.037	0.004	3.033
Scene + pattern + location	0.053	0.002	0.031	0.003	99.897
Scene + pattern + location + Scene × pattern + Scene × location	0.053	6.867 × 10^−4^	0.012	0.001	76.290
Scene + pattern + Scene × pattern	0.053	5.223 × 10^−4^	0.009	9.222 × 10^−4^	70.330
Scene + pattern + location + Scene × location + pattern × location	0.053	2.177 × 10^−4^	0.004	3.845 × 10^−4^	87.934
Scene + pattern	0.053	1.094 × 10^−4^	0.002	1.931 × 10^−4^	54.002
Scene + pattern + location + pattern × location	0.053	7.478 × 10^−6^	1.346 × 10^−4^	1.320 × 10^−5^	92.223
Scene + location	0.053	5.183 × 10^−6^	9.330 × 10^−5^	9.152 × 10^−6^	41.615
Scene + pattern + location + Scene × pattern + Scene × location + pattern × location	0.053	3.021 × 10^−6^	5.438 × 10^−5^	5.335 × 10^−6^	71.873
Scene + pattern + location + Scene × pattern	0.053	1.987 × 10^−6^	3.576 × 10^−5^	3.508 × 10^−6^	57.978
Scene + pattern + location + Scene × pattern + Scene × location + pattern × location + Scene × pattern × location	0.053	1.462 × 10^−7^	2.632 × 10^−6^	2.581 × 10^−7^	97.918
Scene + pattern + location + Scene × pattern + pattern × location	0.053	1.983 × 10^−8^	3.569 × 10^−7^	3.501 × 10^−8^	49.837

## Data Availability

The data presented in this study are openly available at https://zenodo.org/deposit/6815352.
